# Social cognition and social functioning in people with borderline personality disorder and their first-degree relatives

**DOI:** 10.7717/peerj.10212

**Published:** 2020-10-30

**Authors:** Esther Ortega-Díaz, Jonatan García-Campos, José María Rico-Gomis, Carlos Cuesta-Moreno, Antonio Palazón-Bru, Gabriel Estañ-Cerezo, José Antonio Piqueras-Rodríguez, Jesús Rodríguez-Marín

**Affiliations:** 1 Department of Psychiatry, General University Hospital of Elche, Elche, Alicante, Spain; 2 Department of Behavioral and Health Sciences, Miguel Hernández University, San Juan de Alicante, Alicante, Spain; 3 Department of Clinical Medicine, Miguel Hernández University, San Juan de Alicante, Alicante, Spain; 4 Department of Investigation, General University Hospital of Elche, Elche, Alicante, Spain; 5 Department of Health Psychology, Miguel Hernández University, San Juan de Alicante, Alicante, Spain

**Keywords:** Borderline personality disorder, Cognition, Social adjustment, Social behavior, Family

## Abstract

**Background:**

A few papers studying healthy, first-degree relatives of people with borderline personality disorder (BPD) have found that this group presents attention and memory problems. However, current research has not analyzed their social cognition.

**Materials and Methods:**

We designed an age-, gender- and education-level matched case-control study involving 57 people with BPD, 32 of their first-degree relatives, and 57 healthy controls in Spain in 2018–2019. All were assessed for social cognition and functioning using the Movie for Assessment of Social Cognition and the Social Functioning Scale; other potential confounders were also collected (marital status, occupation and household variables).

**Results:**

There were differences in the social cognition domain of overmentalizing errors, with the BPD group scoring significantly higher than controls; however, there was no significant difference with relatives; in the social functioning domain of family relationships, with the controls showing the highest scores. Social engagement/withdrawal, interpersonal behavior, independence-competence, prosocial activities, full scale and categorization domains showed the same pattern: the BPD group had lower scores than their relatives and the controls. Relatives were significantly different from BPD patients in family relationships, social engagement/withdrawal and interpersonal behavior, as well as on the full Social Functioning Scale (both as a linear and categorical variable). However, only controls showed differences with relatives in family relationships.

**Conclusions:**

All in all, relatives show similar levels of social cognition and functioning compared with controls, and people with BPD show some alterations in different domains of both social cognition and functioning.

## Introduction

Borderline personality disorder (BPD) is a severe psychiatric disease that predominantly manifests in young adults through a pattern of instability in interpersonal relationships, self-image, and affect, along with intense impulsivity (American Psychiatric Association, 2014). Epidemiological studies in the United States estimate its prevalence at 0.5–5.9% of the population (Lenzenweger et al., 2007; Grant et al., 2008; Leichsenring et al., 2011)_,_ generating a high burden for public health systems (Soeteman et al., 2008).

Social cognition refers to the abilities to perceive, interpret, and process social stimuli that guide social interactions (Green et al., 2008). Alterations in these processes could cause difficulties in identifying others’ emotions, thoughts, and intentions; these problems could cause different symptoms, such as intense fear of abandonment or dichotomous thinking and idealization (Preißler et al., 2010). Some studies relate the diagnosis of BPD with a disturbance in social cognition (Minzenberg, Poole & Vinogradov, 2006; Preißler et al., 2010), although there is controversy on this point, probably because of the sensitivity of the instruments used (Dziobek et al., 2006; Arntz et al., 2009; Preißler et al., 2010). On the other hand, when more naturalistic methods are used, like the Movie for Assessment of Social Cognition (MASC) (Dziobek et al., 2006), the results more precisely support alterations in the social cognition of people with BPD (Preißler et al., 2010).

Social functioning is a complex and multidimensional construct, encompassing a person’s ability to achieve goals and play defined social roles, as well as to take care of oneself and enjoy leisure time (Mueser & Tarrier, 1998). Some authors note that social functioning covers different areas, including an individual’s social cognition, skills, interactions and behaviors (Beauchamp & Anderson, 2010). In terms of the relationship between these aspects and BDP, people with BPD display lower social functioning compared to the general population (Hill et al., 2008; Gunderson et al., 2011; Liebke et al., 2017), and this difference is even more pronounced in the presence of other psychiatric comorbidities (Mosiołek et al., 2018).

Although few studies have investigated healthy first-degree relatives of people with BPD, these family members present more attention and memory problems than the general population (Ruocco, Lam & McMain, 2014). We have not found studies that analyze social cognition in first-degree relatives of people with BPD, though these studies do exist in other mental pathologies like schizophrenia or bipolar disorder (Lavoie et al., 2013; Reynolds, Van Rheenen & Rossell, 2014). The constant deficit in social cognition has been shown to be a characteristic feature of both of these conditions, extending beyond the period of crisis and constituting an endophenotypic marker in populations with a heightened genetic loading for the disorder, including first-degree relatives (Santos et al., 2017). Moreover, self-perceived function incapacity is increased in both people with BPD and in their first-degree relatives, although in the latter group to a lesser extent and in fewer functional areas (Ruocco, Lam & McMain, 2014).

We are not aware of any research studying social cognition in healthy, first-degree relatives of people with BPD. Thus, our objective was to determine whether diminished social cognition is a characteristic feature in the first-degree relatives of people with BPD.

## Materials and Methods

### Study population

The study included people with BPD, their first-degree relatives (parents or children), and members of the general population without any mental illness, from health department 20 (Valencian Region, in the southeast of Spain). The catchment area of this health department is the entire population of Elche and Santa Pola, which had a registered population of 465,119 inhabitants in 2018 (Conselleria de Sanidad Universal y Salud Pública & Portal del Departamento de Salud de Elche-Hospital General, 2018).

### Study design and participants

This was a case-control study involving 146 participants: 57 with BPD, according to criteria in the fifth version of the *Diagnostic and Statistical Manual of Mental Disorders* (DSM-5) (American Psychiatric Association, 2014); 32 healthy, first-degree relatives; and 57 controls matched with the BPD group for age, gender and educational level. All participants were recruited between July 2018 and March 2019.

People with BPD were chosen from the hospital coding database at the General University Hospital of Elche (the only hospital in the health department); selected patients were those registered with ICD-9-CM code 301.83 and ICD-10-CM code F60.3. Afterwards, a psychiatrist determined whether the patients met the DSM-5 diagnostic criteria (American Psychiatric Association, 2014), and they were contacted by phone to invite them to participate in the study. If the answer was affirmative, they made an appointment at the hospital. Exclusion criteria were: aged under 18 years, another diagnosis of a severe mental disorder, intellectual disability, residence in a different region of Spain, refusal to participate, internment in prison, or inability to contact. First-degree relatives were the BPD participants’ parents or children, who voluntarily agreed to take part in the study; they were excluded if they were younger than 18 years or had a psychiatric disorder (assessed by means of the International Neuropsychiatric Interview; Sheehan et al., 1998). If more than one relative was willing to participate, they were both allowed. On the other hand, if the person with BPD did not have close relatives, if the relative(s) did not want to or could not participate, or if they presented an exclusion criterion, then we collected data only for the person with BPD. The controls were recruited from the companions of patients in the services of surgery, internal medicine, traumatology, neurology, and obstetrics. Selected controls were matched with the BPD study population for age, gender, and educational level. With regard to age, three controls had an age difference of one year with respect to the matched patient. They were assessed using the International Neuropsychiatric Interview to rule out any psychiatric disorders (Sheehan et al., 1998).

### Variables and measurement

Social cognition was measured by means of the Spanish version of MASC (Lahera et al., 2014). This is a naturalistic measure combining auditory, verbal, and emotional channels. For its administration, participants were asked to watch a short film in which four people appeared in different daily situations. During the movie, they had to answer 45 multiple-choice questions about the characters’ feelings, thoughts, and intentions. In addition to the correct answer, there were three error categories: undermentalizing errors, wherein the person has a general—but underdeveloped—idea of what the other could be feeling or thinking; theory of mind absence errors, which occur when there is no connection between one’s observation and interpretation; and overmentalizing errors; which stem from an excessive interpretation of another’s state of mind (Dziobek et al., 2006; Sharp et al., 2011).

The scores took into account both the total number of correct answers and the total errors, and the latter were analyzed by subtype (Lahera et al., 2014). A lower number of correct answers indicates a worse condition. This measure presents high test-retest reliability and high internal consistency in both its original English version (Dziobek et al., 2006) and its translation into Spanish (Lahera et al., 2014).

Social functioning was measured using the Spanish version of the self-administered Social Functioning Scale (SFS, Vázquez & Jiménez, 2000). The SFS assesses seven areas of social functioning in the previous three months on a scale of 70–129: social isolation/integration (0–15), interpersonal communication (0–9), pro-social activities (0–48), recreation (0–32), independence-competence (13–39), independence-performance (0–39), and employment/occupation (0–129). The score cutoffs group respondents in three categories: low-functioning (<95), medium-functioning (95–106), and high-functioning (>106). Two versions of the scale exist, depending on the information source used to understand the patient’s social functioning: in the self-report version, patients themselves complete the questionnaire and provide information on their behavior; and in the informant report, relatives take on this role. Because we aimed to analyze all of the participants’ behavior (people with BPD, relatives and controls), we opted to use the self-report version. The English version shows adequate reliability, validity, and sensitivity (Birchwood et al., 1990). In the Spanish version, internal consistency and test-retest reliability demonstrate some variability, but the results are still satisfactory (Vázquez & Jiménez, 2000).

In addition, the following variables were collected: gender, age (in years), highest educational level attained (primary school, secondary school, and university), marital status (single, married/with stable partner, and separated/widower), occupation (active, unemployed, sick leave/pensioner and student) and household composition (own family, family of origin and single).

Participants were convened in groups of four or fewer in the classroom space at the General University Hospital of Elche. The facilities had a projector, screen, tables, and chairs. Participants received an information sheet on the study and signed informed consent. They then underwent assessments with the MASC and SFS—always in that order and administered by the same professional, a psychiatric occupational therapist with more than 10 years of experience in the service.

### Sample size

The sample size was calculated to compare mean scores on the SFS scale among the three groups (ANOVA). To estimate the means in each group, we randomly selected 15% of the total sample, obtaining the following values: 114.4 for the control group; 115.9 for the relatives; and 101.3 for the BPD group. The estimated standard deviation (SD) was 10.9. According to these parameters and using a type I and type II error of 5%, we calculated a minimum number of 15 participants per group (Chow, Wang & Shao, 2008).

### Statistical methods

Qualitative variables were described as absolute and relative frequencies, while quantitative variables were expressed as means (SD) or medians (interquartile range). To compare group characteristics, Pearson’s chi-squared and ANOVA tests were applied. To assess differences in the scales administered to the three groups, median or ANOVA tests were used, depending on whether the variable of interest was continuous or discrete. Post-hoc analysis was carried out using the Bonferroni correction. For the multivariable analysis, linear or ordinal quantile (median) regression models were fitted to adjust the results for marital status, occupation, and household composition. All analyses were performed at a significance level of 5%, and confidence intervals (CIs) were calculated for each relevant parameter. The statistical software used was IBM SPSS Statistics 25 and R 3.5.1.

### Ethical considerations

Both the Research Commission and the Research Ethics Committee at the General University Hospital of Elche approved the study (25 June 2018 and 26 June 2018, respectively). All participants were adequately informed of the study aims and methods, and if they agreed to take part, they signed informed consent before their inclusion.

## Results

A total of 146 participants were included: 57 had a diagnosis of BPD, 32 were first-degree relatives of these people, and 57 were healthy controls. Tables 1 and 2 show the demographic characteristics of the three groups. Most of the participants in the BPD and control groups were women (91.2%), while a smaller majority were women in the relatives group (62.5%; *p* < 0.001 in the total comparison and *p* = 0.002 when we compared relatives versus controls or patients). Mean age was 33.4 years in the BPD and control groups, and it was 52.9 in the group of first-degree relatives (*p* < 0.001 in both the global analysis and the comparison of relatives with BPD versus controls). Cases and controls showed a similar educational level (*p* > 0.0056), with most having completed secondary school, while the relatives showed a lower level (*p* = 0.003 versus controls and *p* = 0.005 versus patients in secondary school). There were also differences in marital status (*p* = 0.006), occupation (*p* < 0.001), and household composition (*p* = 0.004). Specifically, the relatives were less likely to be single than patients (*p* = 0.002), more likely to be on disability or pension rolls than controls (*p* < 0.001), and less likely to be living with their family of origin compared with patients (*p* = 0.001).

**Table 1:** Sociodemographic factors in the three analyzed groups: people with borderline personality disorder, first-degree relatives and controls.

Variable	Controls	Relatives	BPD	*p*-Value
	*N* = 57	*N* = 32	*N* = 57	
	*n* (%)^†^	*n* (%)^†^	*n* (%)^†^	
Women	52 (91.2)	20 (62.5)	52 (91.2)	<0.001
Educational level				
Primary school	17 (29.8)	19 (59.4)	18 (31.6)	0.027
Secondary school	33 (57.9)	8 (25.0)	32 (56.1)	
University	7 (12.3)	5 (15.6)	7 (12.3)	
Marital status				
Single	18 (31.6)	4 (12.5)	26 (45.6)	0.006
Married/with stable partner	35 (61.4)	20 (62.5)	25 (43.9)	
Separated/widower	4 (7.0)	8 (25.0)	6 (10.5)	
Occupation				
Active	37 (64.9)	12 (37.5)	16 (28.1)	<0.001
Unemployed	5 (8.8)	4 (12.5)	20 (35.1)	
Sick leave/pensioner	3 (5.3)	11 (34.4)	8 (14.0)	
Student	12 (21.1)	5 (15.6)	13 (22.8)	
Household composition				
Own family	40 (70.2)	25 (78.1)	24 (42.1)	0.004
Family of origin	13 (22.8)	4 (12.5)	22 (38.6)	
Single	4 (7.0)	3 (9.4)	11 (19.3)	
Age (years), mean ± SD	33.4 ± 10.7	52.9 ± 16.3	33.4 ± 10.7	<0.001

**Notes:**

† Unless otherwise noted.

BPD, borderline personality disorder; SD, standard deviation.

**Table 2:** Post-hoc analysis with the Bonferroni correction (*p*-values) of the sociodemographic factors in the three analyzed groups: people with borderline personality disorder, first-degree relatives and controls.

Variable	Relatives vs. controls	Relatives vs. BPD	BPD vs. controls	Number of comparisons	Significance (<*p*-value)
Women	0.002	0.002	>0.999	3	0.017
Educational level Primary school Secondary school University	0.006 0.003 0.750	0.011 0.005 0.750	0.839 0.850 >0.999	9	0.0056
Marital status Single Married/with stable partner Separated/widower	0.045 0.919 0.024	0.002 0.091 0.072	0.124 0.061 0.508	9	0.0056
Occupation Active Unemployed Sick leave/pensioner Student	0.013 0.717 <0.001 0.532	0.358 0.021 0.025 0.418	<0.001 <0.001 0.113 0.821	12	0.0042
Household composition Own family Family of origin Single	0.417 0.235 0.699	0.001 0.009 0.217	0.003 0.068 0.052	9	0.0056
Age	<0.001	<0.001	>0.999	3	0.017

**Note:**

BPD, borderline personality disorder.

Bivariable analysis of the questionnaires among the three groups (Tables 3 and 4) showed statistically significant differences (*p* < 0.05) in the number of correct MASC items (*p* = 0.012) and the MASC Overmentalizing errors (*p* = 0.006). Controls scored higher on the first and lower on the second; relatives did not present statistical differences with the other groups (*p* > 0.017). All SFS dimensions showed differences as well (*p* < 0.05), with the relatives group presenting higher scores than the BPD group. However, the only dimensions reaching statistical differences between relatives and BPD patients (*p* < 0.017) were social engagement/withdrawal (*p* < 0.001), independence-performance (*p* = 0.008) and recreation (*p* = 0.008). In contrast, relatives showed significantly higher overall scores on the SFS than BPD patients (*p* < 0.001) and similar scores to the control group (*p* = 0.198). When the variable was categorized, likewise there was a significantly larger proportion of relatives compared to BPD patients with higher scores (*p* = 0.001).

**Table 3:** Scores of the scales applied in the three study groups.

Item	Controls *N* = 57	Relatives *N* = 32	BPD *N* = 57	*p*-Value
MASC, median (IQR)				
Correct	31 (6)	27 (9)	28 (5)	0.012
Overmentalizing errors	6 (4)	8 (6)	8 (4)	0.006
Undermentalizing errors	6 (3)	6 (4)	6 (3)	0.23
Theory of mind absence errors	2 (3)	3 (2)	3 (3)	0.27
SFS domains, median (IQR)				
Family relationships	10 (2)	9 (2)	7 (3)	<0.001
Social engagement/withdrawal	13 (2)	12 (2)	9 (5)	<0.001
Interpersonal behavior	8 (1)	8 (1)	7 (3)	**^†^**
Independence-performance	34 (8)	32 (10)	28 (10)	0.004
Independence-competence	39 (0)	38 (1)	36 (4)	**^†^**
Recreation	22 (8)	22 (8)	18 (7)	0.009
Prosocial activities	23 (11)	20 (12)	16 (16)	0.011
Employment/occupation	9 (1)	9 (1)	6 (8)	<0.001
SFS full scale, mean ± SD	114.5 ± 5.5	112.6 ± 8.4	102.4 ± 11.6	<0.001
SFS scores, *n* (%)				
Low	0 (0)	2 (6.3)	12 (21.1)	<0.001
Medium	5 (8.8)	4 (12.5)	20 (35.1)	
High	52 (91.2)	26 (81.3)	25 (43.9)	

**Notes:**

† It was not possible to carry out the median test due to the number of valid cases.

Abbreviations: BPD, borderline personality disorder; MASC, movie for the assessment of social cognition; IQR, interquartile range; SFS, social functioning scale.

**Table 4:** Post-hoc analysis with the Bonferroni Correction (*p*-values) for the scores of the scales applied in the three study groups.

Item	Relatives vs. controls	Relatives vs. BPD	BPD vs. controls	Number of comparisons	Significance (<*p*-value)
MASC					
Correct	0.080	0.887	0.005	3	0.017
Overmentalizing errors	0.347	0.842	0.002	3	0.017
SFS domains					
Family relationships	0.021	0.055	<0.001	3	0.017
Social engagement/withdrawal	0.709	<0.001	<0.001	3	0.017
Independence-performance	0.832	0.008	<0.001	3	0.017
Recreation	0.104	0.008	0.005	3	0.017
Pro-social activities	0.142	0.236	0.003	3	0.017
Employment/occupation	**^†^**	0.148	<0.001	3	0.017
SFS full scale	0.198	<0.001	<0.001	3	0.017
SFS scores				9	0.0056
Low	0.127	0.066	<0.001		
Medium	0.717	0.021	0.001		
High	0.193	0.001	<0.001		

**Notes:**

† It was not possible to carry out the median test due to the number of valid cases.

Abbreviations: BPD, borderline personality disorder; MASC, movie for the assessment of social cognition; SFS, social functioning scale.

Table 5 shows the results of the multivariable analysis, assessing differences between groups in the questionnaires used, but adjusting for marital status, occupation, and household composition. After controlling for these confounders, using the relatives group as a reference, there were no significant differences in the MASC subscales. On the family relationships subscale of the SFS, the controls scored the highest (2, *p* < 0.001), followed by the relatives (0) and finally the cases (−2, *p* = 0.014). The SFS subscales of social engagement/withdrawal, interpersonal behavior, independence-competence, and prosocial activities, plus the full scale and the categorization, showed the same pattern, with the BPD group achieving lower scores than the relatives and the controls. However, the statistical differences (*p* < 0.05) were in: social engagement/withdrawal (relatives vs. BPD, *p* = 0.002), interpersonal behavior (relatives vs. BPD, *p* = 0.019), SFS full scale (relatives vs. BPD, *p* < 0.001) and categorized SFS scores (relatives vs. BPD, *p* = 0.003).

**Table 5:** Multivariable analysis of the scales used in our patients, relatives and controls (coefficients with their 95% confidence intervals).

Variable	Relatives	Controls	*p*-Value	BPD	*p*-Value
Quantile regression					
MASC Correct Overmentalizing errors Undermentalizing errors Theory of mind absence errors	0 0 0 0	1 (−3 to 5) −1 (−4 to 2) 0 (−2 to 2) −0.5 (−2 to 1)	0.634 0.457 >0.999 0.561	0 (−3 to 3) 1 (−2 to 4) −1 (−3 to 1) 0 (−2 to 2)	>0.999 0.506 0.250 >0.999
SFS domains Family relationships Social engagement/withdrawal Interpersonal behavior Independence-performance Independence-competence Recreation Prosocial activities Employment/occupation	0 0 0 0 0 0 0 0	2 (1 to 3) 0 (−1 to 1) 0 (0 to 0) 0 (−3 to 3) 0 (0 to 0) −2 (−6 to 2) 1 (−6 to 8) 0 (−1 to 1)	<0.001 >0.999 >0.999 >0.999 >0.999 0.283 0.778 >0.999	−2 (−4 to 0) −2.5 (−4 to −1) −1 (−2 to 0) −3 (−7 to 1) −2 (−4 to 0) −4 (−8 to 0) −5 (−12 to 2) −1 (−3 to 1)	0.014 0.002 0.019 0.132 0.052 0.083 0.189 0.319
Linear regression					
SFS (full scale)	0	−0.94 (−4.7 to 2.8)	0.627	−9.9 (−13.7 to −6.1)	<0.001
Ordinal regression (odds ratio)					
SFS (categorized)	1	1.24 (0.27 to 5.76)	0.780	0.14 (0.04 to 0.52)	0.003

**Notes:**

BPD, borderline personality disorder; MASC, movie for the assessment of social cognition; SFS, social functioning scale.

All the coefficients were adjusted by marital status, occupation and household composition.

## Discussion

### Summary

The present study investigated social cognition and functioning in people with BPD, their healthy, first-degree relatives, and a group of healthy controls. Our results show that relatives of people with BPD show some alterations in social cognition; however, results were not statistically significant, so there is insufficient evidence to support that this is a characteristic feature of BPD. With regard to social functioning, first-degree relatives showed a significant deficit on the family relationships subscale compared to controls and BPD patients, and in social engagement/withdrawal and interpersonal behavior compared with patients. We observed similar results when assessing the SFS full scale, as both a linear and categorical variable.

### Strengths and limitations

The main strength of our study is its novel nature; we did not find any other paper in the literature examining social cognition in first-degree relatives of people with BPD. In addition, the statistical power was over 95% for testing differences in means in the calculation of the sample size, which increases the precision of our results.

To minimize selection bias, the sampling frame was the list of patients in the hospital coding database, not those attending the consult, as in previous studies (Minzenberg, Poole & Vinogradov, 2006). This is an important issue, as it increases the representativeness of the sample of people with BPD. Furthermore, the controls were selected using a population-based approach from the same geographical area, and they did not have any other pathology, especially mental disorders. With regard to information bias, data collection was undertaken by a single professional with experience administering the questionnaires used in this study, which enhances the reliability of the results obtained. Other studies have not taken the same precautions to limit this form of bias (Lahera et al., 2014). In addition, we used internationally validated scales; MASC (Dziobek et al., 2006) is much more naturalistic and precise than other measurement instruments. Moreover, by estimating the magnitude of effects through multivariable models, we could minimize the risk of confounding bias, as evidenced by the loss of statistical significance between the bivariable and the multivariable analysis after adjusting for other factors. On the other hand, it was not feasible to match the relatives for gender, age, or educational level, therefore we cannot rule out the influence of confounding in this group. Furthermore, we could not guarantee that the patients with BPD were in the same disease stage during assessment, which could alter the results and should be taken into account in future studies. Finally, we were unable to determine whether the origin of the alterations of function and the perception of emotions were due to genetic or environmental factors, for example, living with a person that had BPD.

### Comparison with existing literature

We did not find any article that assessed social cognition in first-degree family members of people with BPD, although there are similar studies in other mental disorders, like bipolar disorder and schizophrenia, that have reported alterations. In the case of schizophrenics, their relatives did not show important deficits in social cognition, but they did show lower performance than the general population (Lavoie et al., 2013; Reynolds, Van Rheenen & Rossell, 2014). These results, together with the literature reporting that alterations in social cognition can be observed in patients “in remission” (Bora, Yucel & Pantelis, 2009) support the hypothesis that social cognition capacities may be related to a disorder’s genetic component (Gottesman & Gould, 2003), and deficits in these processes could stem from genetic vulnerability in BPD (Lavoie et al., 2013; Reynolds, Van Rheenen & Rossell, 2014).

Our results differ from those of other studies that have not found any diminishment of social cognition in people with BPD, for example, in Preißler et al. (2010) study, which used the “Reading the Mind in the Eyes” test (RME), or Arntz et al. (2009) paper, which used the advanced test of theory of mind. On the other hand, Minzenberg, Poole & Vinogradov (2006) used the Buss-Durkee hostility index, finding a normal capacity for recognizing isolated facial or prosodic emotions but difficulties in recognizing integrated ones. These differences could be due to the psychometric tools used, as naturalistic scales like MASC (Dziobek et al., 2006) yield more precise results. Other groups that have used MASC, like Preißler et al. (2010) and Sharp et al. (2011), have reported similar results to ours, with BPD patients showing alterations in social cognition. We obtained higher scores in social cognition in the form of overmentalization errors, which coincides with Sharp et al. (2011) study in a sample of adolescents with borderline features.

With regard to social functioning, our results differ from Ruocco, Lam & McMain (2014), where relatives reported greater functional limitations than controls in life activities and participation in society. However, in our study, despite the lower scores achieved by relatives compared to controls in some domains, significant differences were only apparent in family relationships. Likewise, we found small but significant differences in our BPD group in the domains of social engagement/withdrawal, interpersonal behavior, independence-competence, prosocial activities, and on the full-scale SFS, similarly to Ruocco, Lam & McMain (2014), who reported that the probands showed higher levels of incapacity than their relatives and the controls in all functional domains: comprehension and communication, mobility, self-care, interpersonal relations, life activities (domestic, leisure, work and academic activities), and participation in society. Finally, Liebke et al. (2017) used the same social functioning scale as we did, although their sample did not include first-degree relatives. Patients with BPD showed low social functioning, while in our sample they presented medium functioning. Likewise, in their study there were significant alterations in all domains in BPD participants compared to controls, while in our study the differences were not significant in the domains of recreation or employment/occupation. These differences could be due to the distinct cultural characteristics, as their study took place in Germany, while ours was in Spain. Finally, Skodol et al. (2005) used the Longitudinal Interval Follow-up Evaluation, and they found significant deficits in the domains of interpersonal behavior, prosocial activities, full scale and occupation. However, it is difficult to draw a comparison with our results because of the different measures used.

### Implications for clinical practice and research

We detected diminished social cognition skills in people with BPD, along with limitations in some domains of social functioning in both the people with BPD and in their first-degree relatives. These results could support development of interventions to reduce the deficits identified. In light of our findings, future studies are needed to determine whether a deficit in the domain of family relationships in healthy relatives influences the social functioning, social cognition, and/or the symptomology of people with BPD. Additional research is also needed to understand the pathophysiology of BPD, including the role of genetic and socioenvironmental factors.

## Conclusions

Our results show that healthy first-degree relatives of people with BPD present similar social cognition skills as healthy controls, with no genetic vulnerability related to BPD. The social cognition of people with this disorder demonstrates greater deficits in the form of overmentalization. Compared to patients, relatives showed significant differences in social functioning with regard to family relationships, social engagement/withdrawal and interpersonal behavior, and compared to controls, relatives showed differences in family relationships. Otherwise, social functioning is quite similar between relatives and controls, while people with BPD show lower social functioning across many domains.

## Supplemental Information

10.7717/peerj.10212/supp-1 Supplemental Information 1 Raw data: the measurements in the three analyzed groups.

## Additional Information and Declarations

### Data Availability

The following information was supplied regarding data availability: Raw data are available as a Supplemental File.
